# Exploring the Cardiovascular Benefits of Sodium-Glucose Cotransporter-2 (SGLT2) Inhibitors: Expanding Horizons Beyond Diabetes Management

**DOI:** 10.7759/cureus.46243

**Published:** 2023-09-30

**Authors:** Aroob Fatima, Sohaib Rasool, Sapna Devi, Muhammad Talha, Fahad Waqar, Muhammad Nasir, Mohammad R Khan, Syed M Ibne Ali Jaffari, Anum Haider, Syeda U Shah, FNU Sapna, Giustino Varrassi, Mahima Khatri, Satesh Kumar, Tamam Mohamad

**Affiliations:** 1 Medicine, Fatima Memorial Hospital College of Medicine and Dentistry, Lahore, PAK; 2 Medicine, Bakhtawar Amin Medical and Dental College, Multan, PAK; 3 Internal Medicine, Ziauddin University, Karachi, PAK; 4 Internal Medicine, Nishtar Medical University, Multan, PAK; 5 Medicine, Allama Iqbal Medical College, Lahore, PAK; 6 Medicine, Rural Health Center (RHC) Dhonkal, Dhonkal Morr, PAK; 7 Internal Medicine, Bakhtawar Amin Trust Teaching Hospital, Multan, PAK; 8 Medicine and Surgery, Shalamar Medical and Dental college, Lahore, PAK; 9 Internal Medicine, International Medical Graduates Helping Hand, Karachi, PAK; 10 Medical College, Jinnah Sindh Medical University, Karachi, PAK; 11 Pathology, Albert Einstein College of Medicine, Bronx, USA; 12 Pain Medicine, Paolo Procacci Foundation, Rome, ITA; 13 Medicine and Surgery, Dow University of Health Sciences, Karachi, PAK; 14 Medicine and Surgery, Shaheed Mohtarma Benazir Bhutto Medical College, Karachi, PAK; 15 Cardiovascular Medicine, Wayne State University, Detroit, USA

**Keywords:** sglt2 inhibitors, heart failure management, renoprotection, cardioprotection, diabetes management, cardiovascular benefits

## Abstract

Globally, cardiovascular disease (CVD) continues to be the primary cause of morbidity and mortality. The risk of cardiovascular disease is markedly increased in individuals with type 2 diabetes mellitus (T2DM), making managing cardiovascular health a top priority. Initially developed for their glucose-lowering properties, sodium-glucose cotransporter 2 (SGLT2) inhibitors have emerged as a transformative class of pharmaceuticals with profound cardiovascular benefits that extend far beyond glycemic control. One of the most striking findings is the substantial reduction in major adverse cardiovascular events (MACE), including myocardial infarction, stroke, and cardiovascular mortality, observed in clinical trials evaluating SGLT2 inhibitors. These extraordinary cardioprotective effects are demonstrated by landmark trials such as EMPA-REG OUTCOME, CANVAS, and DECLARE-TIMI 58, which are discussed in detail. In addition, SGLT2 inhibitors have demonstrated positive outcomes in heart failure (HF) with reduced ejection fraction, which has led to their incorporation into HF treatment guidelines. SGLT2 inhibitors offer renoprotection by delaying the progression of diabetic kidney disease, reducing albuminuria, preserving glomerular filtration rates, and their immediate cardiovascular benefits. We investigate the potential mechanisms underlying these renal benefits, focusing on the role of hemodynamic alterations and intraglomerular pressure reduction. In addition, SGLT2 inhibitors have a distinct diuretic effect that can contribute to volume reduction and symptom alleviation in patients with heart failure (HF). This diuretic action, distinct from conventional diuretics, warrants additional research to optimize their use in T2DM and HF patients. The risk of euglycemic diabetic ketoacidosis, genital mycobacterial infections, and bone fractures are also discussed. Understanding these issues is essential for making educated clinical decisions. In conclusion, SGLT2 inhibitors have transcended their initial function as anti-diabetic agents to become essential components of cardiovascular and renal protection strategies in T2DM patients. Their diverse benefits, which include cardioprotection, renoprotection, and the potential for HF management, highlight their potential to transform cardiovascular medicine. Optimizing the use of SGLT2 inhibitors in clinical practice bears the promise of improved cardiovascular outcomes for patients with T2DM and beyond as we navigate this changing landscape.

## Introduction and background

Diabetes mellitus, a chronic metabolic disorder characterized by high blood glucose levels, poses a significant threat to global health. Type 2 diabetes mellitus (T2DM) is the most prevalent type, accounting for about 90% of all cases of diabetes worldwide. To prevent complications such as cardiovascular disease, kidney disease, and neuropathy, T2DM must be effectively managed. For the treatment of T2DM, sodium-glucose cotransporter 2 (SGLT2) inhibitors have emerged as a novel and promising class of drugs. This review seeks to provide an in-depth analysis of SGLT2 inhibitors, their mechanisms of action, clinical applications, and potential benefits, with a particular emphasis on their impact on glycemic control, cardiovascular outcomes, renal protection, and safety. SGLT2 inhibitors are a relatively new addition to the arsenal of anti-diabetes drugs. They inhibit SGLT2, a protein found primarily in the kidney's proximal renal tubules. SGLT2's primary function is to reabsorb glucose from the urine back into the circulation, thus contributing to hyperglycemia in T2DM patients. This glucose reabsorption mechanism is inhibited by SGLT2 inhibitors, such as canagliflozin, dapagliflozin, and empagliflozin, resulting in increased urinary glucose excretion and decreased blood glucose levels [1]. This insulin-independent mechanism of action makes SGLT2 inhibitors suitable as monotherapy or in combination with other antidiabetic agents [2].

T2DM is a global health epidemic with a continuously increasing prevalence. According to the International Diabetes Federation (IDF), more than 463 million persons around the world had diabetes in 2019, and this number is expected to rise to 700 million by 2045. T2DM significantly contributes to the global disease burden, highlighting the need for effective and innovative treatment options [2]. Inhibitors of SGLT2 represent a paradigm shift in treating type 2 diabetes. Traditional diabetes medications predominantly target insulin secretion or insulin sensitivity. In contrast, SGLT2 inhibitors provide a novel mechanism of action by promoting glucose excretion independent of insulin [3]. This novel approach has garnered great interest from healthcare professionals, researchers, and patients, necessitating a comprehensive analysis of their full potential. Beyond glucose control, SGLT2 inhibitors have demonstrated a wide range of clinical benefits. These advantages include body mass index, blood pressure, and cardiovascular outcomes. In addition, SGLT2 inhibitors have renoprotective effects, which are especially important for T2DM patients at risk for diabetic nephropathy and chronic kidney disease [4]. The exploration and analysis of these multifaceted effects requires a comprehensive review. While SGLT2 inhibitors offer numerous advantages, they are not without safety concerns. Among the potential adverse effects are genital infections, urinary tract infections, and rare but severe conditions such as diabetic ketoacidosis [5].

A comprehensive review of the safety profile is essential for healthcare providers to optimize the risk-benefit ratio when prescribing these medications. The primary objective of this review is to provide a comprehensive and current analysis of SGLT2 inhibitors in the treatment of T2DM. The particular goals are as follows: this review will assess the efficacy of SGLT2 inhibitors in treating T2DM. Among the most important efficacy parameters to be evaluated are their effects on glycemic control, as measured by decreases in HbA1c and fasting blood glucose levels. Their use as monotherapy and in combination with other antidiabetic agents will be investigated. Cardiovascular disease is the primary cause of morbidity and mortality in individuals with T2DM [5]. Therefore, we will comprehensively analyze the cardiovascular benefits of SGLT2 inhibitors, including their effects on major adverse cardiovascular events (MACE) and heart failure outcomes. Understanding their impact on cardiovascular health is vital, given the high risk of T2DM. SGLT2 inhibitors have shown promise in protecting renal function and delaying the progression of chronic kidney disease in patients with type 2 diabetes [6]. We will explore the renal benefits of these medications and their prospective implications for diabetic nephropathy and CKD patients. An important aspect of the clinical profile of SGLT2 inhibitors [6] is their effect on body weight. We will investigate the magnitude of weight loss attained with these medications and their function in combating obesity, a prevalent comorbidity of T2DM. In evaluating any medication, safety is of paramount importance. This analysis will evaluate the safety profile of SGLT2 inhibitors, including potential adverse effects and their management. We will offer guidance on minimizing risks and maximizing benefits. It is essential to summarize and present the current clinical guidelines and recommendations regarding the use of SGLT2 inhibitors in T2DM management to guide clinical practice. This section will give healthcare professionals insights into implementing these agents in clinical practice. This review seeks to provide a comprehensive understanding of SGLT2 inhibitors, including their mechanism of action, clinical efficacy, cardiovascular and renal benefits, safety profile, and clinical considerations for healthcare providers. By addressing these objectives, we hope to contribute to the body of knowledge surrounding these medications, thereby enhancing the care and outcomes for T2DM patients.

## Review

Methods

Formation of Research Question

The first stage in conducting this narrative review was formulating a precise research question: "What is the breadth and depth of the cardiovascular benefits of SGLT2 inhibitors, and how do they transcend their conventional role in diabetes management?" This research question directs the selection of relevant literature, data extraction, synthesis, and analysis throughout the narrative review.

Strategy for Literature Search Databases

A systematic and exhaustive literature survey was conducted to identify relevant studies and publications. The following databases were used to ensure that a broad spectrum of biomedical and clinical literature was covered: PubMed, an essential resource for biomedical literature; Scopus, a multidisciplinary database with extensive coverage of scientific journals; Web of Science, which provides access to a vast array of research domains; and Embase, a comprehensive database specializing in pharmacology and biomedical literature. This selection of databases ensured that a broad range of scholarly works were included in the review.

Keywords and key phrases: To establish an efficient search strategy, a comprehensive list of keywords and search terms were compiled, including "SGLT2 inhibitors", "cardiovascular benefits", "heart failure", "atherosclerosis", "renal outcomes", and "mechanisms of action." These terms were combined in various methods using Boolean operators (AND, OR) to encompass all topic aspects. For example, "SGLT2 inhibitors AND cardiovascular benefits" emphasized the cardiovascular benefits of SGLT2 inhibitors.

Inclusion and exclusion requirements: To facilitate the selection of articles, explicit inclusion and exclusion criteria were formulated. The inclusion criteria stipulated that studies must investigate the cardiovascular effects of SGLT2 inhibitors beyond diabetes management. Studies not published in English or unrelated to the research query were excluded based on exclusion criteria. These criteria were rigorously followed during the selection process to ensure the relevance of the chosen studies.

Search filters: Databases' search filters were utilized to refine search results further. These filters permitted the restriction of search results by publication date, study type, and other relevant criteria. For example, studies published within the last decade were frequently prioritized to ensure the review included the most recent findings. In addition to the electronic database searches, a thorough manual search was conducted by scrutinizing the reference lists of identified articles and pertinent reviews; this manual search aimed to identify any additional studies that may have eluded the initial electronic search.

Selection of Studies

Following the completion of the literature search, the following steps were performed to select applicable studies:

Screening: A preliminary review of the search results was conducted based on the predetermined inclusion and exclusion criteria. The titles and abstracts of articles were evaluated extensively to identify potentially relevant studies.

Full-text evaluation: The full texts of potentially relevant articles were analyzed in detail to determine their compatibility with the research question and objectives. Articles that contributed to the comprehension of the cardiovascular benefits of SGLT2 inhibitors and met the inclusion criteria were selected for inclusion in the review. Relevant data were extracted from the selected studies in a systematic manner. This included information regarding the study's design, patient demographics, interventions, outcomes, and significant findings. Data extraction was performed meticulously to ensure the inclusion of all pertinent details.

Synthesis and Analysis of Data

The procedure of narrative review included synthesis and analysis of the extracted data. The organization of findings according to themes, study categories, or pertinent subtopics allowed for a coherent narrative presentation. A comprehensive analysis of the available evidence was conducted to discern patterns, relationships, and variations in the cardiovascular benefits of SGLT2 inhibitors. The research query guided the synthesis, which aimed to understand the topic comprehensively.

Quality Assessment

Using appropriate methods and criteria, the quality and rigor of the included studies were assessed. Study design, sample size, methodological rigor, and potential biases were evaluated to determine the evidence's validity. This quality evaluation allowed for consideration of the strengths and weaknesses of the selected studies and their impact on the overall narrative review. This exhaustive methodology ensured a systematic and rigorous approach to investigating the cardiovascular benefits of SGLT2 inhibitors beyond their traditional function in managing diabetes, providing a solid foundation for the narrative review.

Mechanisms of action

Sodium-glucose cotransporter 2 (SGLT2) inhibitors, a class of anti-diabetic drugs, have emerged as productive glucose-lowering agents and multifaceted therapeutic agents with significant cardiovascular and renal benefits [3]. Understanding the mechanisms underlying their actions is essential to recognizing their clinical significance. This in-depth analysis examines the mechanisms of action of SGLT2 inhibitors, including their impact on glucose regulation and hemodynamic effects, supported by pertinent scientific literature.

Overview of the Action Mechanisms of SGLT2 Inhibitors

The SGLT2 protein in the proximal renal tubules of the kidneys is the primary target of SGLT2 inhibitors. This protein is responsible for glucose reabsorption from glomerular filtrate into the circulation. By inhibiting SGLT2, these drugs reduce renal glucose reabsorption, increasing urinary glucose excretion, a condition known as glycosuria. This mechanism is crucial to their glucose-lowering effect [5].

**Figure 1:**
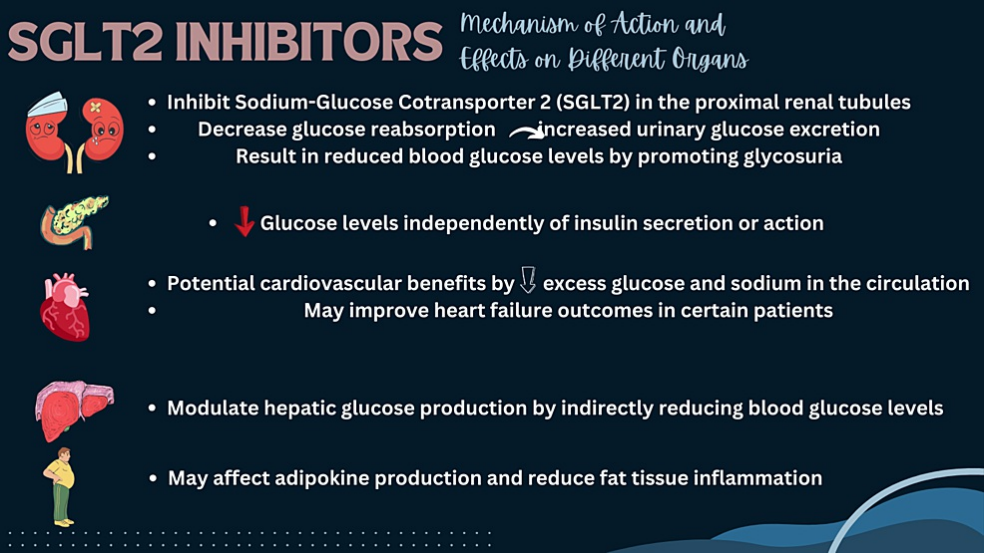
Mechanism of action of SGLT2 inhibitors on different organ systems Image credits: Satesh Kumar, Mahima Khatri SGLT2 - sodium-glucose cotransporter 2

Influence on glucose control: SGLT2 inhibitors provide a novel and efficient method of glucose regulation. They reduce blood sugar levels by encouraging the excretion of excess glucose in the urine. This effect is independent of insulin and is frequently associated with a decreased risk of hypoglycemia, a common side effect of other diabetes medications. In addition, SGLT2 inhibitors exhibit an insulin-independent mode of action, rendering them suitable for use in patients with varying degrees of insulin resistance and type 2 diabetes mellitus (T2DM) [5]. Their efficacy in lowering HbA1c levels is well-documented, with clinical trials demonstrating significant decreases when used alone or combined with other anti-diabetic agents [6]. Beyond their immediate effect on glycemic control, SGLT2 inhibitors have been shown to positively affect various metabolic health parameters. They contribute to weight loss due predominantly to the caloric loss associated with increased glucose excretion in the urine. This weight loss can be especially advantageous for overweight or obese T2DM patients [7]. In addition, SGLT2 inhibitors enhance insulin sensitivity and beta-cell function, improving glucose regulation [7]. This multidimensional approach to glycemic management highlights their significance in treating type 2 diabetes. Figure 1 highlights the multifaceted effects of SGLT2 inhibitors on various organ systems, emphasizing their role in managing type 2 diabetes and potentially improving cardiovascular and renal health.

Physiological effects: The mechanisms of action of SGLT2 inhibitors extend far beyond glycemic control, with significant implications for cardiovascular and renal health. The reduction in blood pressure is a notable hemodynamic effect. [8] Numerous studies have demonstrated that SGLT2 inhibitors cause modest but clinically significant reductions in systolic and diastolic blood pressure. However, due to increased glucose excretion, natriuresis and osmotic diuresis are likely to be involved. The enhancement of arterial rigidity and endothelial function is another crucial hemodynamic effect. The efficacy of SGLT2 inhibitors to improve vascular health by reducing arterial stiffness, an independent predictor of cardiovascular events, has been demonstrated. Improved endothelial function contributes to vasodilation, decreasing vascular resistance and blood pressure [8]. SGLT2 inhibitors have also been linked to decreased intraglomerular pressure within the kidneys. These medications can potentially delay the progression of diabetic kidney disease by reducing the glomerular load. Decreased intraglomerular pressure may also contribute to maintaining glomerular filtration rate (GFR) in patients with impaired renal function [9]. Inhibitors of SGLT2 have demonstrated significant benefits in the management of heart failure. It has been demonstrated that they reduce the risk of hospitalization for heart failure and alleviate symptoms in patients with T2DM and heart failure, regardless of diabetes. This effect is attributable to their diuretic effect, reduction in preload and afterload, and prospective impact on myocardial energy metabolism [7]. In addition, SGLT2 inhibitors have been linked to beneficial effects on lipid profiles, including reductions in triglycerides and increases in high-density lipoprotein (HDL) cholesterol [10, 11]. Together with their weight-reducing effects, these lipid modifications contribute to a more cardioprotective metabolic profile. In conclusion, the action mechanisms of SGLT2 inhibitors extend far beyond their principal function in glucose regulation. Their effect on glucose excretion, blood pressure reduction, vascular health, and the management of heart failure exemplifies their central role in enhancing cardiovascular and renal outcomes in patients with T2DM and beyond. These multifaceted effects highlight the increasing significance of SGLT2 inhibitors as a cornerstone in managing diabetes and associated complications, establishing them as transformative agents in contemporary medicine.

Cardiovascular outcomes in diabetes

Cardiovascular disease (CVD) is intricately related to diabetes mellitus, a metabolic disorder characterized by chronic hyperglycemia. Understanding the cardiovascular risk associated with diabetes and the urgent need for cardiovascular protection in diabetic patients is essential for comprehensive patient care and better clinical outcomes [11]. This in-depth investigation examines the intricate relationship between diabetes and cardiovascular outcomes, highlighting the critical need for effective cardiovascular risk mitigation strategies in this high-risk population. Diabetes and the risk of cardiovascular disease diabetes, especially type 2 diabetes mellitus (T2DM), is a well-established independent risk factor for cardiovascular disease. Multiple pathophysiological mechanisms, including insulin resistance, hyperglycemia, dyslipidemia, inflammation, and endothelial dysfunction, underlie the association between diabetes and CVD [12]. Insulin resistance, a defining characteristic of T2DM, promotes atherogenic alterations in lipid metabolism and contributes to the development of atherosclerosis. Insulin resistance can also result in hypertension, a significant risk factor for cardiovascular disease. In diabetes, prolonged hyperglycemia can result in microvascular complications like retinopathy, nephropathy, and neuropathy. It also accelerates atherosclerosis and increases the risk of macrovascular complications, including coronary artery disease (CAD), stroke, and peripheral arterial disease (PAD) [13]. Elevated triglycerides, decreased high-density lipoprotein (HDL) cholesterol, and increased small, dense, low-density lipoprotein (LDL) particles characterize diabetic dyslipidemia. This lipid profile contributes to the risk of cardiovascular disease [10]. Chronic inflammation, a prevalent characteristic of diabetes, serves a crucial role in the pathogenesis of atherosclerosis. Inflammatory markers, such as C-reactive protein (CRP) and interleukin-6 (IL-6), are associated with an increased risk of cardiovascular disease (CVD) events [14]. Diabetes impairs endothelial function, resulting in decreased vasodilation, increased vasoconstriction, and a prothrombotic condition. These modifications increase the likelihood of atherosclerosis and thrombotic events [13].

Need for Cardiovascular Protection in Patients with Diabetes

Given the substantial cardiovascular risk associated with diabetes, diabetic patients must implement effective cardiovascular protection strategies. Diabetes is the primary cause of morbidity and mortality due to cardiovascular disease. Patients with diabetes are two to four times more likely to die from cardiovascular disease than those without diabetes. CVD tends to occur younger among people with diabetes, increasing the number of years of prospective life lost due to cardiovascular events [11]. Diabetes frequently coexists with other cardiovascular risk factors, such as hypertension and dyslipidemia, exacerbating the risk of cardiovascular disease [14]. Diabetic patients who experience a cardiovascular event, such as a heart attack or stroke, tend to have worse outcomes, higher rates of complications, and increased healthcare costs than non-diabetic individuals [15]. Several approaches, including lifestyle modifications, pharmacological interventions, and targeted management strategies, have been created to address the critical need for cardiovascular protection in diabetic patients. Lifestyle interventions focusing on a healthy diet, regular physical activity, smoking cessation, and weight management are fundamental for diabetes management and cardiovascular protection. Medications targeting major cardiovascular risk factors, such as statins for dyslipidemia and antihypertensive agents for blood pressure control, play a central role in reducing the risk of cardiovascular disease (CVD) risk in people with diabetes [16]. Aspirin or other antiplatelet agents may be prescribed in some instances to reduce the risk of thrombus formation and cardiovascular events, particularly in people with diabetes at high risk. Beyond glycemic control, novel anti-diabetic agents, such as sodium-glucose cotransporter 2 (SGLT2) inhibitors and glucagon-like peptide-1 (GLP-1) receptor agonists, have demonstrated substantial cardiovascular benefits. These agents are now regarded as integral to the management of type 2 diabetes and the reduction of cardiovascular risk [17]. Integrating the management of diabetes and cardiovascular risk factors into comprehensive care models provides a holistic approach to cardiovascular protection. This includes routine monitoring, risk assessment, and customized treatment plans. The relationship between diabetes and cardiovascular complications is complex and multifaceted. Diabetes significantly increases the risk of cardiovascular disease and is a primary cause of morbidity and mortality among those affected. Comprehensive cardiovascular protection strategies, including lifestyle modifications, pharmacological interventions, and novel anti-diabetic agents such as SGLT2 inhibitors and GLP-1 receptor agonists, are required to address this crucial issue. These strategies are essential for reducing the prevalence of CVD among diabetic patients and enhancing overall clinical outcomes.

Cardioprotective effects of SGLT2 inhibitors

Cardiovascular diseases (CVDs) are a leading cause of worldwide morbidity and mortality. In recent years, there has been a paradigm shift in managing type 2 diabetes mellitus (T2DM) due to the advent of sodium-glucose cotransporter 2 (SGLT2) inhibitors. Initially developed for glycemic control, these drugs have shown remarkable cardioprotective effects beyond glucose control. This discussion delves into the cardioprotective effects of SGLT2 inhibitors, mainly focusing on the reduction in major adverse cardiovascular events (MACE) as demonstrated in pivotal clinical trials, including EMPA-REG OUTCOME, CANVAS, and DECLARE-TIMI 58. We will also explore the underlying mechanisms responsible for these beneficial cardiovascular effects.

Reduction in Major Adverse Cardiovascular Events (MACE): Insights from Clinical Trials

EMPA-REG OUTCOME trial: The EMPA-REG OUTCOME trial was a landmark study that assessed the cardiovascular outcomes of empagliflozin, an SGLT2 inhibitor. It involved over 7,000 patients with T2DM and established CVD. The trial demonstrated a 14% relative risk reduction in the composite primary endpoint of MACE, which included cardiovascular death, non-fatal myocardial infarction (MI), and non-fatal stroke, over a median follow-up of 3.1 years [17]. Furthermore, empagliflozin was associated with a 38% relative risk reduction in cardiovascular death, a remarkable finding that underscored the potential cardioprotective effects of SGLT2 inhibitors.

CANVAS program: The CANVAS program evaluated canagliflozin, another SGLT2 inhibitor, in patients with T2DM and a high risk of CVD. This program demonstrated a 14% reduction in the risk of MACE, mainly driven by a reduction in the risk of non-fatal MI [18]. Interestingly, canagliflozin also reduced heart failure hospitalizations, an effect not previously seen with anti-diabetic agents.

DECLARE-TIMI 58 trial: The DECLARE-TIMI 58 trial examined dapagliflozin in a broader population of patients with T2DM, including those without established CVD. Although the primary endpoint of MACE reduction was not met, there was a significant reduction in hospitalization for heart failure [19]. This finding suggested that SGLT2 inhibitors may substantially impact heart failure outcomes, particularly in high-risk individuals.

Mechanisms Underlying Cardioprotection

The mechanisms by which SGLT2 inhibitors confer cardioprotection are not fully understood but are likely multifactorial.

Improved glycemic control: SGLT2 inhibitors primarily lower blood glucose levels by inhibiting renal glucose reabsorption, leading to glycosuria. Improved glycemic control can contribute to reduced microvascular complications, which, in turn, may positively impact cardiovascular outcomes [20].

Hemodynamic effects: SGLT2 inhibitors have been shown to reduce blood pressure, arterial stiffness, and preload through natriuresis and osmotic diuresis [21]. These effects can alleviate the workload on the heart, reducing the risk of cardiac events.

Reduced myocardial injury: Animal studies suggest that SGLT2 inhibitors may protect the heart from ischemia-reperfusion injury by modulating mitochondrial function, reducing oxidative stress, and improving myocardial energetics [22]. SGLT2 inhibitors can promote a shift from glucose to ketone bodies as a fuel source, which may provide more efficient energy production in the myocardium, especially during stress [23]. Emerging evidence suggests that SGLT2 inhibitors may have direct anti-inflammatory and anti-fibrotic effects on the myocardium, reducing adverse cardiac remodeling [24]. SGLT2 inhibitors are associated with weight loss, which can positively impact cardiovascular risk factors such as insulin resistance and inflammation [25]. Some studies have suggested that SGLT2 inhibitors can improve endothelial function, potentially reducing atherosclerotic progression [26].

In conclusion, SGLT2 inhibitors have emerged as a transformative class of anti-diabetic agents with remarkable cardioprotective effects. Clinical trials, including EMPA-REG OUTCOME, CANVAS, and DECLARE-TIMI 58, have consistently shown reductions in MACE and heart failure hospitalizations in patients with T2DM, with or without established CVD. While the precise mechanisms underlying these benefits are still being elucidated, SGLT2 inhibitors likely exert their effects through a combination of improved glycemic control, hemodynamic effects, myocardial protection, metabolic shifts, and anti-inflammatory actions. These findings have revolutionized the management of T2DM and expanded our understanding of the complex interplay between diabetes, cardiovascular disease, and metabolic pathways. Further research is needed to explore these drugs' long-term safety and efficacy in diverse patient populations and uncover additional mechanisms responsible for their cardioprotective effects. SGLT2 inhibitors have opened new avenues for preventing and managing cardiovascular complications in individuals with T2DM, offering hope for improved patient outcomes and reduced healthcare burden.

Heart failure management

Heart failure (HF) is a prevalent and life-threatening cardiovascular condition characterized by the heart's inability to pump blood efficiently, leading to symptoms like breathlessness, fatigue, and fluid retention. It is a primary global health concern, affecting millions worldwide, with significant morbidity and mortality rates [27]. The management of heart failure is multifaceted, involving lifestyle modifications, pharmacological interventions, and, in some cases, surgical procedures or device therapy. Among the various therapeutic approaches, sodium-glucose cotransporter 2 (SGLT2) inhibitors have emerged as a novel and promising class of drugs in the treatment of heart failure with reduced ejection fraction (HFrEF) [20]. This article aims to delve into the role of SGLT2 inhibitors in HFrEF management, drawing from clinical trials that have demonstrated their benefits and exploring potential mechanisms behind these improvements.

Role of SGLT2 Inhibitors in Heart Failure with Reduced Ejection Fraction (HFrEF)

SGLT2 inhibitors, initially developed for the management of type 2 diabetes mellitus, have shown remarkable benefits in heart failure management, particularly in patients with reduced ejection fraction (HFrEF), unlike traditional HF medications like beta-blockers and ACE inhibitors, which primarily target neurohormonal pathways, SGLT2 inhibitors work by a different mechanism [11]. They inhibit glucose reabsorption in the renal tubules, increasing urinary glucose excretion. However, their effects on HFrEF extend beyond glycemic control.

Clinical Trials Demonstrating Benefits in HFrEF

Multiple clinical trials have investigated the efficacy of SGLT2 inhibitors in patients with HFrEF. Among the most influential trials are the DAPA-HF and EMPEROR-Reduced trials, which have demonstrated significant benefits in reducing HF-related morbidity and mortality. The Dapagliflozin and Prevention of Adverse Outcomes in Heart Failure (DAPA-HF) trial was a landmark study that assessed the effects of dapagliflozin, an SGLT2 inhibitor, in HFrEF patients with or without type 2 diabetes. The trial enrolled 4,744 patients and found that dapagliflozin reduced the risk of the composite primary outcome (a cardiovascular death, hospitalization for HF, or an urgent visit for HF) by 26%, compared to the placebo group [28]. The EMPEROR-Reduced trial investigated empagliflozin, another SGLT2 inhibitor, in 3,730 HFrEF patients. The trial demonstrated that empagliflozin significantly reduced the risk of cardiovascular death and hospitalization for HF by 25%, compared to the placebo group [29]. These trials provided strong evidence for the beneficial effects of SGLT2 inhibitors in HFrEF patients, leading to their incorporation into heart failure guidelines and clinical practice.

Potential Mechanisms for Improved Heart Failure Outcomes

The precise mechanisms behind the improvements seen with SGLT2 inhibitors in HFrEF are still under investigation. However, several plausible mechanisms have been proposed: SGLT2 inhibitors induce osmotic diuresis by promoting glucose excretion in the urine. This leads to a reduction in intravascular volume and cardiac preload, alleviating congestion and symptoms of HF [30]. SGLT2 inhibitors have improved myocardial energetics by enhancing myocardial glucose utilization and reducing fatty acid oxidation. This shift in substrate utilization may improve cardiac efficiency and function [31]. SGLT2 inhibitors can lower blood pressure and reduce arterial stiffness, potentially reducing the workload on the heart and improving cardiac function [32]. Recent studies suggest that SGLT2 inhibitors may exert natriuretic and anti-fibrotic effects, reducing cardiac fibrosis and inflammation, vital contributors to HF progression [33]. SGLT2 inhibitors have been associated with reduced left ventricular hypertrophy and improved cardiac structure and function, possibly through their effects on myocardial and vascular remodeling [34]. The management of heart failure, especially in patients with reduced ejection fraction, has witnessed a significant paradigm shift with the introduction of SGLT2 inhibitors. Clinical trials such as DAPA-HF and EMPEROR-Reduced have unequivocally demonstrated their efficacy in reducing cardiovascular events and hospitalizations in HFrEF patients. Although the precise mechanisms behind these benefits are still being elucidated, the diuretic effect, improvements in myocardial energetics, blood pressure reduction, and potential anti-fibrotic effects are among the proposed mechanisms. Incorporating SGLT2 inhibitors into heart failure guidelines has changed the landscape of HFrEF management. Future research will likely focus on optimizing their use, identifying patient populations that may benefit the most, and exploring additional mechanisms through which these drugs can improve heart failure outcomes.

Renoprotection and diabetic kidney disease

Diabetic kidney disease (DKD), a common complication of diabetes, is a leading cause of chronic kidney disease (CKD) and end-stage renal disease (ESRD) worldwide. Managing DKD is crucial to mitigate the risk of kidney failure and improve overall patient outcomes. Sodium-glucose cotransporter 2 (SGLT2) inhibitors, initially developed for glycemic control in diabetes, have garnered significant attention for their potential to offer renoprotection beyond glycemic management [33]. This article provides a detailed exploration of SGLT2 inhibitors' impact on DKD, renal outcomes in clinical trials, and the underlying mechanisms of renoprotection. SGLT2 inhibitors' impact on diabetic kidney disease (DKD) The role of SGLT2 inhibitors in DKD management has gained prominence due to their ability to address multiple pathophysiological pathways involved in the progression of renal disease. These agents primarily target the SGLT2 transporters in the proximal renal tubules, inhibiting glucose reabsorption and promoting glucosuria [12].

Clinical Trials Demonstrating Renal Outcomes

Several landmark clinical trials have assessed the renoprotective effects of SGLT2 inhibitors in patients with DKD. Two pivotal trials that have contributed substantially to our understanding of their renal benefits are: the Canagliflozin and Renal Events in Diabetes with Established Nephropathy Clinical Evaluation (CREDENCE) trial evaluated canagliflozin, an SGLT2 inhibitor in patients with type 2 diabetes and established DKD. This trial revealed that canagliflozin reduced the risk of the primary composite endpoint (end-stage kidney disease, doubling serum creatinine, renal or cardiovascular death) by 30% compared to the placebo group [30]. The Dapagliflozin and Prevention of Adverse Outcomes in Chronic Kidney Disease (DAPA-CKD) trial investigated dapagliflozin's effects on renal outcomes in patients with CKD, including those without diabetes. Dapagliflozin significantly reduced the risk of the primary composite endpoint (sustained decline in estimated glomerular filtration rate, end-stage kidney disease, or renal or cardiovascular death) by 39% compared to the placebo group [35].

Mechanisms Underlying Renoprotection

The mechanisms underlying the renoprotective effects of SGLT2 inhibitors in DKD are multifaceted and continue to be explored. Some proposed mechanisms include SGLT2 inhibitors, which reduce intraglomerular pressure and hyperfiltration by promoting natriuresis and reducing sodium reabsorption. This effect on glomerular hemodynamics helps alleviate the strain on the renal vasculature and slows the progression of renal damage [35]. SGLT2 inhibitors have consistently shown a substantial reduction in albuminuria, a hallmark of DKD. This reduction may be attributed to their ability to decrease glomerular pressure and inflammation and their impact on the tubuloglomerular feedback mechanisms [36]. Emerging evidence suggests that SGLT2 inhibitors possess anti-inflammatory and anti-fibrotic properties. They may reduce oxidative stress, inflammation, and fibrosis within the renal parenchyma, thereby preserving kidney function [33]. SGLT2 inhibitors lead to weight loss and improve metabolic parameters, including blood pressure and lipid profiles. These metabolic improvements can indirectly contribute to renal protection by reducing cardiovascular risk factors [34]. SGLT2 inhibitors may protect podocytes, specialized cells in the renal glomerulus, from injury. Podocyte preservation can help maintain the integrity of the glomerular filtration barrier and reduce proteinuria [35]. Diabetic kidney disease significantly burdens individuals with diabetes, often leading to chronic kidney disease and end-stage renal disease. Sodium-glucose cotransporter 2 (SGLT2) inhibitors have emerged as a promising therapeutic option for managing DKD, with evidence from clinical trials such as CREDENCE and DAPA-CKD demonstrating their substantial renal benefits. The renoprotective effects of SGLT2 inhibitors are multifactorial, with mechanisms including improved glomerular hemodynamics, reduced albuminuria, anti-inflammatory and anti-fibrotic properties, metabolic improvements, and podocyte protection. These mechanisms collectively contribute to the slowing of renal disease progression. As our understanding of SGLT2 inhibitors' role in DKD continues to evolve, ongoing research will likely focus on optimizing their use, identifying patient populations that benefit the most, and elucidating additional mechanisms contributing to renal protection.

Diuretic effects and fluid management

Heart failure (HF) is a complex cardiovascular condition characterized by the heart's inability to pump blood efficiently, leading to fluid accumulation, congestion, and reduced cardiac output. Diuretic therapy has been a cornerstone in managing HF symptoms by promoting the removal of excess fluid. Sodium-glucose cotransporter 2 (SGLT2) inhibitors, initially developed for diabetes management, have emerged as novel therapeutics with diuretic properties [37]. This article explores the diuretic effects of SGLT2 inhibitors, their implications for volume management in heart failure, and how they compare with traditional diuretics.

Diuretic Properties of SGLT2 Inhibitors

SGLT2 inhibitors, such as dapagliflozin, empagliflozin, and canagliflozin, primarily target the renal proximal tubules to reduce glucose reabsorption. In inhibiting glucose reabsorption, these drugs lead to natriuresis and increased urinary excretion of glucose and sodium [38]. While SGLT2 inhibitors were initially developed to lower blood glucose levels in diabetes, their mechanism of action in the kidneys extends beyond glycemic control. By inhibiting glucose and sodium reabsorption in the proximal tubules, SGLT2 inhibitors induce natriuresis, resulting in increased urinary sodium excretion. This sodium loss contributes to osmotic diuresis, enhancing fluid removal [20]. The primary mechanism by which SGLT2 inhibitors induce diuresis is osmotic diuresis. Increased glucose excretion in the urine generates an osmotic gradient that prevents water reabsorption in the renal tubules, promoting diuresis and fluid elimination [31].

Implications for Volume Management in Heart Failure

The diuretic effects of SGLT2 inhibitors carry significant implications for managing volume overload in heart failure patients, especially those with reduced ejection fraction (HFrEF). Fluid overload and congestion are hallmark features of HF. SGLT2 inhibitors can alleviate congestion by promoting diuresis and reducing intravascular volume. This reduction in congestion leads to symptom relief, including improved dyspnea and reduced peripheral edema [23]. By efficiently removing excess fluid and relieving congestion, SGLT2 inhibitors may decrease HF-related hospitalizations. This reduction in hospitalizations is a critical endpoint in HF management, as it improves patients' quality of life and reduces healthcare costs [34]. SGLT2 inhibitors can complement the action of traditional diuretics like loop diuretics (e.g., furosemide). Combining these agents may allow for lower doses of loop diuretics, reducing the risk of electrolyte imbalances and renal dysfunction associated with high-dose diuretic therapy [35]. While SGLT2 inhibitors exhibit diuretic properties, comparing their effects with traditional diuretics used in HF management, such as loop diuretics and thiazides, is essential. SGLT2 inhibitors induce diuresis through osmotic mechanisms, resulting in sustained and gradual fluid removal.

In contrast, loop diuretics often lead to rapid diuresis but may require higher doses and pose a greater risk of electrolyte imbalances [36]. SGLT2 inhibitors have potential renal benefits, such as preserving the glomerular filtration rate (GFR) and reducing albuminuria. Traditional diuretics, particularly loop diuretics at high doses, may be nephrotoxic and adversely affect renal function over time [37]. SGLT2 inhibitors have demonstrated cardiovascular benefits in addition to their diuretic effects, including reduced cardiovascular mortality and hospitalizations. Traditional diuretics primarily address volume overload without similar cardiovascular outcomes [38]. Sodium-glucose cotransporter 2 (SGLT2) inhibitors, initially developed for diabetes management, have emerged as promising agents with diuretic properties in heart failure management. Their unique mechanism of action in the renal tubules induces natriuresis and osmotic diuresis, leading to efficient fluid removal and congestion relief. These diuretic effects have significant implications for volume management in heart failure, potentially reducing hospitalizations and improving patient outcomes. Compared to traditional diuretics, SGLT2 inhibitors offer sustained diuresis, potential renal protection, and additional cardiovascular benefits. Combining these agents with traditional diuretics may optimize fluid management while minimizing the risk of adverse effects associated with high-dose diuretic therapy. As research in this area continues, SGLT2 inhibitors are poised to play an increasingly prominent role in the comprehensive management of heart failure, addressing fluid overload and cardiovascular outcomes.

Safety considerations

Sodium-glucose cotransporter 2 (SGLT2) inhibitors have revolutionized the management of type 2 diabetes mellitus by effectively lowering blood glucose levels through a unique mechanism of inhibiting glucose reabsorption in the renal tubules. While these medications have shown remarkable benefits in improving glycemic control and cardiovascular outcomes, they also have specific safety considerations. This article explores three significant safety concerns associated with SGLT2 inhibitors: the risk of euglycemic diabetic ketoacidosis (DKA), genital mycotic infections, and bone fractures. We will investigate these issues, examining the potential mechanisms and providing insights into their management.

Euglycemic Diabetic Ketoacidosis (DKA) Risk

Euglycemic diabetic ketoacidosis (DKA) is a rare but potentially life-threatening complication of SGLT2 inhibitors. Unlike classic diabetic ketoacidosis (DKA), euglycemic (eDKA) occurs without significantly elevated blood glucose levels, often presenting with near-normal or only mildly elevated glucose levels [38]. The mechanisms behind eDKA in SGLT2 inhibitor users are not fully understood. Still, they are believed to involve several factors: SGLT2 inhibitors can lead to increased glucagon secretion, promoting hepatic ketogenesis even when blood glucose levels are not substantially elevated [39]. These medications induce osmotic diuresis, increasing urination and potential dehydration, exacerbating ketoacidosis. SGLT2 inhibitors may reduce insulin secretion or sensitivity, contributing to an insufficient insulin response in the presence of rising ketone levels [40]. To mitigate the risk of eDKA, healthcare providers should carefully assess patients before initiating SGLT2 inhibitor therapy. Patients with a history of DKA, pancreatitis, severe dehydration, or other risk factors may not be suitable candidates for these medications [38]. Additionally, it is crucial to educate patients about the signs and symptoms of DKA, such as nausea, vomiting, abdominal pain, and altered mental status, so they can seek immediate medical attention if necessary [39].

Genital Mycotic Infections

Genital mycotic infections, particularly genital yeast infections, are everyday adverse events associated with SGLT2 inhibitor use. These infections can cause discomfort and inconvenience for patients. SGLT2 inhibitors increase the glucose content in the urinary tract, creating a favorable environment for fungal overgrowth [35]. Additionally, the glucose-rich genital secretions may promote the growth of Candida species, leading to infections. To manage and prevent genital mycotic infections, patients should be advised to maintain proper genital hygiene and consider the use of topical antifungal agents for symptomatic relief [34]. In some cases, discontinuing the SGLT2 inhibitor or switching to an alternative medication may be necessary if recurrent or severe infections occur.

Increased Risk of Bone Fractures

Emerging evidence suggests a potential association between SGLT2 inhibitor use and an increased risk of bone fractures, particularly in older individuals. The mechanisms underlying this association are not yet fully elucidated. Still, several hypotheses have been proposed: SGLT2 inhibitors may affect bone mineral density by altering calcium and phosphate homeostasis in the body. These medications can increase urinary calcium excretion, potentially affecting bone health [25]. Some patients experience weight loss while taking SGLT2 inhibitors, which could impact bone health, especially in those already at risk for fractures [30]. Healthcare providers should consider the individual patient's fracture risk when prescribing SGLT2 inhibitors. Patients at high risk for fractures, such as the elderly or those with a history of fractures, may require closer monitoring and possibly alternative treatments. Adequate calcium and vitamin D intake and weight-bearing exercise should also be encouraged to support bone health [29]. SGLT2 inhibitors have transformed the management of type 2 diabetes mellitus and offer numerous benefits in terms of glycemic control and cardiovascular outcomes. However, healthcare providers and patients need to be aware of potential safety considerations associated with these medications. Euglycemic diabetic ketoacidosis, genital mycotic infections, and bone fractures are among the notable safety concerns. While these issues are relatively rare and manageable, healthcare providers should carefully assess patients' suitability for SGLT2 inhibitor therapy, educate them about potential risks, and monitor their response during treatment [30]. As ongoing research sheds light on these safety considerations, a more comprehensive understanding of the mechanisms and effective management strategies will further enhance the safe and beneficial use of SGLT2 inhibitors in clinical practice.

Practical considerations and clinical guidelines

Sodium-glucose cotransporter 2 (SGLT2) inhibitors have emerged as a valuable class of medications for managing type 2 diabetes mellitus (T2DM). These drugs offer unique mechanisms of action beyond glycemic control, including cardiovascular benefits, weight reduction, and potential renal protection. However, the successful use of SGLT2 inhibitors in clinical practice necessitates adherence to clinical guidelines and thoughtful consideration of patient-specific factors [33]. This comprehensive discussion will explore practical considerations and clinical guidelines for using SGLT2 inhibitors, covering clinical recommendations, patient selection criteria, and monitoring strategies.

Clinical Recommendations for SGLT2 Inhibitor Use

Clinical guidelines and recommendations for using SGLT2 inhibitors in diabetes management have evolved, incorporating evidence from landmark trials. Key clinical recommendations include: SGLT2 inhibitors are recommended as first-line therapy in patients with T2DM and established cardiovascular disease (CVD) to reduce the risk of major adverse cardiovascular events (MACE) [2]. SGLT2 inhibitors are strongly recommended for patients with T2DM and heart failure (HF), particularly those with reduced ejection fraction (HFrEF), to reduce HF hospitalizations and cardiovascular mortality [2]. SGLT2 inhibitors are recommended for patients with T2DM and chronic kidney disease (CKD) to slow the progression of renal impairment, regardless of whether they have heart failure [34]. SGLT2 inhibitors can be considered part of the treatment regimen for T2DM patients requiring additional glucose lowering. However, they may not be the preferred initial choice in patients without specific indications [30].

Patient Selection Criteria

To maximize the benefits and minimize potential risks, patient selection for SGLT2 inhibitor therapy should consider several factors: Patients with established cardiovascular disease, heart failure, or chronic kidney disease, especially those with albuminuria, stand to gain substantial benefits from SGLT2 inhibitors [2]. Despite other therapies, SGLT2 inhibitors are generally appropriate for T2DM patients with suboptimal glycemic control. However, carefully evaluating individual glycemic targets and preferences is essential [26]. Assess the patient's risk factors for adverse events, including euglycemic diabetic ketoacidosis (eDKA), genital mycotic infections, and bone fractures. Patients with DKA or recurrent genital infections may need alternative therapies [2]. Consider the patient's preferences and lifestyle when selecting SGLT2 inhibitors. Dosing frequency, tolerability, and potential weight loss effects may influence treatment choices [3].

Monitoring and Follow-Up

Monitoring and follow-up are critical components of safe and effective SGLT2 inhibitor therapy: Regularly monitoring glycemic control with hemoglobin A1c (HbA1c) and self-monitoring of blood glucose (SMBG). Adjusting therapy as needed to achieve individualized glycemic targets. Assessing renal function at baseline and periodically during treatment, including estimated glomerular filtration rate (eGFR) and urinary albumin-to-creatinine ratio (UACR). Adjusting drug dosages or consider discontinuation based on eGFR changes. Monitoring blood pressure regularly, as SGLT2 inhibitors may lead to reductions in systolic and diastolic blood pressure. Adjusting antihypertensive medications accordingly. Monitoring weight and assessing for fluid balance regularly, as SGLT2 inhibitors can lead to diuresis and weight loss [38]. Addressing concerns related to dehydration or orthostatic hypotension. Educating patients about the signs and symptoms of eDKA, such as nausea, vomiting, abdominal pain, and altered mental status. Encouraging immediate medical attention if these symptoms occur. Informing patients about the increased risk of genital mycotic infections and guide hygiene and antifungal treatments. Addressing any recurrent or severe infections promptly [36]. Assessing the patient's fracture risk, particularly in older individuals. Encouraging weight-bearing exercise, calcium and vitamin D supplementation, and considerations for alternative therapies if necessary. Providing comprehensive patient education about the benefits, risks, and potential side effects of SGLT2 inhibitors. Empowering patients to engage in their diabetes management actively.

Sodium-glucose cotransporter 2 (SGLT2) inhibitors have become valuable therapeutic options for managing type 2 diabetes mellitus [37]. Clinical guidelines and recommendations highlight their role in improving cardiovascular outcomes, heart failure management, and renal protection. However, the safe and effective use of SGLT2 inhibitors necessitates thoughtful consideration of patient-specific factors. Patient selection criteria should be based on individual cardiovascular and renal health, glycemic control, safety considerations, and patient preferences [39]. Monitoring and follow-up are crucial for optimizing therapy and addressing potential adverse events. Healthcare providers are pivotal in educating patients, facilitating shared decision-making, and ensuring that SGLT2 inhibitors are integrated into comprehensive diabetes care plans. As research continues to uncover additional benefits and safety considerations, the clinical landscape of SGLT2 inhibitor use in diabetes management will evolve, offering even more excellent opportunities to improve patient outcomes.

Future directions and research gaps

Sodium-glucose cotransporter 2 (SGLT2) inhibitors have transformed the landscape of type 2 diabetes management by providing not only effective glycemic control but also cardiovascular and renal benefits. As these medications continue to gain prominence in clinical practice, ongoing research is shedding light on their expanding role in various disease states and uncovering potential applications beyond diabetes. In this comprehensive discussion, we will delve into the future directions and research gaps in SGLT2 inhibitors, including ongoing studies, potential clinical indications, and the unanswered questions that warrant further investigation.

Ongoing Research in SGLT2 Inhibitors

Ongoing research endeavors are exploring the multifaceted effects of SGLT2 inhibitors and their implications in diverse medical conditions. Several notable areas of ongoing research include clinical trials, such as EMPEROR-Preserved and EMPEROR-Reduced, which investigate the efficacy of SGLT2 inhibitors in patients with heart failure with preserved ejection fraction (HFpEF) and heart failure with reduced ejection fraction (HFrEF), respectively [39, 40]. These trials aim to expand the evidence base for SGLT2 inhibitors' role in heart failure management. Trials like DAPA-CKD are examining the renal benefits of SGLT2 inhibitors in patients with chronic kidney disease, including those without diabetes [20]. This research aims to elucidate the potential renoprotective effects in a broader patient population. SGLT2 inhibitors have shown promise in reducing hepatic fat content and improving liver function markers in individuals with NAFLD [23]. Ongoing studies are further exploring their potential in managing this prevalent liver condition. Research is underway to evaluate the role of SGLT2 inhibitors in obesity management, as they can induce weight loss through caloric loss via glucosuria and potential appetite reduction [25]. These studies may lead to novel approaches for obesity treatment. Ongoing trials are investigating the cardiovascular benefits of SGLT2 inhibitors in various patient populations, including those with heart failure, acute coronary syndromes, and primary prevention [31, 32]. These studies aim to refine our understanding of their cardiovascular effects.

Potential Expansions of Clinical Indications

The expanding clinical indications for SGLT2 inhibitors hold promise for a broader range of patients beyond those with diabetes: SGLT2 inhibitors have already received approval for heart failure management in patients with or without diabetes. Future guidelines may incorporate these medications as a standard of care in heart failure treatment protocols. SGLT2 inhibitors may become a pivotal therapy for slowing the progression of chronic kidney disease, independently of diabetes status, especially in individuals with albuminuria or those at high renal risk. If ongoing research confirms their efficacy, SGLT2 inhibitors could become a therapeutic option for non-alcoholic fatty liver disease (NAFLD) and its more severe form, non-alcoholic steatohepatitis (NASH). SGLT2 inhibitors may find a place in obesity management strategies, offering weight loss benefits that complement dietary and lifestyle interventions. Further research may lead to the consideration of SGLT2 inhibitors in primary prevention for individuals at high risk of cardiovascular events, potentially expanding their use in a preventive context.

Unanswered Questions and Areas Requiring Further Investigation

While SGLT2 inhibitors have shown immense potential, several unanswered questions and areas requiring further investigation remain: The precise mechanisms underlying SGLT2 inhibitors' cardiovascular and renal benefits are not fully elucidated. Continued research into their molecular and physiological effects is essential for a comprehensive understanding. The long-term safety of SGLT2 inhibitors, especially in diverse patient populations, needs extensive evaluation. Further research should focus on potential rare adverse events and their management. Identifying the optimal patient profiles and criteria for initiating SGLT2 inhibitor therapy in different clinical contexts is an ongoing challenge. More research is needed to refine patient selection criteria. Research into the safety and efficacy of combining SGLT2 inhibitors with other diabetes medications, such as GLP-1 receptor agonists, insulin, and traditional oral agents, can provide insights into optimal combination strategies. Assessing the cost-effectiveness of SGLT2 inhibitors in various healthcare settings and economic contexts is crucial for guiding healthcare policies and reimbursement decisions. Studies that capture real-world data on the use of SGLT2 inhibitors, including patient adherence, persistence, and outcomes, can offer valuable insights into their practical clinical impact. SGLT2 inhibitors have redefined the landscape of diabetes management by offering cardiovascular, renal, and potentially broader health benefits. Ongoing research continues to unveil their multifaceted effects and expanding clinical applications. As we move forward, addressing unanswered questions, refining patient selection criteria, and assessing long-term safety will be paramount in optimizing the use of these medications in clinical practice. The future of SGLT2 inhibitors promises to extend their reach beyond diabetes to various medical conditions, ultimately enhancing patient care and improving outcomes across a spectrum of diseases.

## Conclusions

Clinical guidelines now recommend SGLT2 inhibitors for specific patient groups, leading to transformative changes in managing type 2 diabetes. These medications offer a practical means of controlling blood sugar levels while providing vital cardiovascular and renal protection. They have firmly established themselves as the go-to treatment for patients with diabetes, underlying cardiovascular issues, heart failure, or chronic kidney disease. Ongoing research suggests promising applications in conditions beyond diabetes, such as obesity and non-alcoholic fatty liver disease (NAFLD). As the role of SGLT2 inhibitors continues to evolve, healthcare providers must remain attentive to emerging evidence and adapt their clinical approaches accordingly. These drugs represent a new frontier in healthcare, showcasing the potential of targeted therapies to address intricate, interconnected health conditions, ultimately leading to improved patient outcomes and a better quality of life.
